# Postprocessing Method for Enhanced Left Ventricular Thrombus Detection in Echocardiography

**DOI:** 10.3390/medicina60111815

**Published:** 2024-11-05

**Authors:** Marina Leitman, Vladimir Tyomkin

**Affiliations:** 1 Cardiology Department, Shamir Medical Center, Zerifin 707300, Israel; 2 Sackler School of Medicine, Tel Aviv University, Tel Aviv 6997801, Israel

**Keywords:** left ventricular thrombus detection method, thrombus in the left ventricle, apical thrombus, echocardiography

## Abstract

*Background and Objectives:* The incidence of left ventricular thrombus has decreased in recent years due to advancements in reperfusion strategies for acute myocardial infarction and the use of medications to reduce ventricular remodeling. However, the accurate detection of thrombus remains crucial. Echocardiography is a primary diagnostic tool for thrombus detection, but in cases where the apex of the left ventricle is not clearly visualized, contrast injection is often required for diagnosis. We developed a postprocessing Left Ventricular Thrombus Detection Method (LVTDM) to enhance image details in the region of interest, enabling diagnosis without additional contrast injection. A purpose of our study is the evaluation of Left Ventricular Thrombus Detection Method. *Materials and Methods:* We analyzed echocardiography video files from 29 patients with apical wall motion abnormalities using LVTDM to identify the presence or absence of thrombus in the left ventricular apex. The results were verified with diagnoses obtained from the same echocardiography examinations following contrast injection. Our method demonstrated a sensitivity of 100% and a specificity of 83%, with a negative predictive value of 100% for ruling out thrombus. There was a strong correlation in thrombus detection/ruling out between LVTDM and contrast echocardiography. The Left Ventricular Thrombus Detection Method can be integrated into routine echocardiography examinations to effectively rule out thrombus when the apex is not clearly visualized. The implementation of this method has the potential to reduce the need for contrast injection by approximately half for detecting left ventricular thrombus.

## 1. Introduction

Thrombus formation in the left ventricle following myocardial infarction, particularly in cases of anterior infarction, has decreased due to improved reperfusion strategies and medications designed to prevent left ventricular remodeling. However, the risk of thrombus formation remains significant, affecting more than 9% of these patients [1]. Moreover, in cases of dilated cardiomyopathy, the incidence of thrombus formation can rise to as high as 36% [2]. Additionally, patients with hypertrophic apical cardiomyopathy are at risk of developing thrombus, particularly in the presence of apical aneurysms [3]. In the general population, the risk of thrombus detection is 7 per 10,000 patients [4]. The presence of a left ventricular thrombus carries a substantial risk of embolization, estimated at 22% [5]. Transthoracic echocardiography is the standard imaging modality for detecting thrombus. The administration of contrast during echocardiography has been shown to significantly enhance the sensitivity of left ventricular thrombus detection, potentially doubling the detection rate [5]. As a result, detecting left ventricular thrombus is a primary indication for contrast echocardiography [6]. However, the routine use of contrast may not be necessary when high-quality echocardiographic images are obtained. In this study, we developed a software method to enhance left ventricular visualization during conventional transthoracic echocardiography, especially in cases of suboptimal image quality. The method aims to maximize the utility of detailed information from standard echocardiographic images. We applied this approach to a cohort of patients for whom conventional echocardiography alone was insufficient, requiring contrast injection for definitive diagnosis. Our method utilizes the Reinhard Tone Mapping Operator (TMO) to improve visualization and details in the apical region of the heart without the need for contrast agents. Reducing the reliance on contrast injections is especially important for patients with renal dysfunction or those at risk of allergic reactions. In this study, we present our findings and the correlations observed with contrast echocardiography.

## 2. Materials and Methods

Echocardiography examinations performed between 2023 and 2024, in which the contrast agent SonoVue was administered for the diagnosis of left ventricular thrombus, were identified. The pre-contrast injection video files were then subjected to postprocessing using our proposed Left Ventricular Thrombus Detection Method (LVTDM). The goal was to enhance the quality of these echocardiography images to a diagnostic level. Both the original pre-contrast and post-LVTDM video files were encoded for analysis. Two independent observers, blinded to the patient data and the stage of postprocessing, analyzed all 58 encoded videos. Each video was assessed for the presence or absence of thrombus, or classified as non-diagnostic. The observers then compared their results, and the correlation with videos obtained after the injection of SonoVue was subsequently analyzed.

### 2.1. Inclusion Criteria

Use of SonoVue Contrast Agent: The study included patients whose echocardiography required the use of the SonoVue contrast agent to diagnose thrombus in the left ventricle.

Echocardiography Examinations with Suboptimal Image Quality: Patients whose initial echocardiography was suboptimal, leading to the use of contrast agents, were included to test the efficacy of LVTDM.

Age: Adult patients (≥18 years) to focus on an adult population at risk of thrombus formation.

The patients were enrolled consecutively.

### 2.2. Exclusion Criteria

Non-Diagnostic Echocardiography Post-Contrast Injection: Echocardiography examinations where, even after the use of contrast agents, a definitive diagnosis could not be made were excluded.

### 2.3. Why We Need Left Ventricular Thrombus Detection Method

Technically challenging echocardiographic images often hinder accurate diagnosis. During transmission to the workstation, the quality of echocardiography videos may degrade, and current image enhancement techniques—such as compression, rejection, and persistence adjustments—available on echocardiography systems or viewers, typically offer only slight improvements. These methods are often insufficient for making an accurate diagnosis and, when improperly applied, may further reduce image quality. While contrast injection can improve visualization, it carries risks of allergic reactions, is problematic in patients with renal failure, adds time, and incurs additional costs. We propose a postprocessing method, LVTDM, which works on already recorded echocardiographic videos, maximizing the available information without requiring contrast agents. Our method enhances the diagnostic accuracy, especially in cases where image quality is suboptimal.

### 2.4. Postprocessing: Left Ventricular Thrombus Detection Method

The Left Ventricular Thrombus Detection Method aims to maximize the information that can be obtained from echocardiography videos. LVTDM employs a known tone mapping technique typically used for converting high dynamic range images into a format suitable for display on standard monitors with lower dynamic range. In the context of echocardiography videos with limited dynamic range, this technique is adapted to enhance diagnostic details specifically in the apical area, which serves as the region of interest.

LVTDM selectively increases the range of mid-tones within the apical region, enhancing diagnostic details while maintaining appropriate contrast and clarity. This targeted adjustment aims to improve the visualization of potential thrombus formations within the apex of the left ventricle, particularly in cases where conventional echocardiography images may lack clarity or sufficient quality.

During the postprocessing phase, minor smoothing of details occurs in areas outside of the region of interest, particularly in high-intensity pixel areas. This ensures that while enhancing diagnostic details within the apical region, the overall image quality and clarity are preserved throughout the echocardiography video.

We found that the best results were achieved with Reinhard Tone Mapping Operator (TMO) [7]. This operator employs a straightforward formula for image conversion:

*Iout* = *Iin*/(*Iin* + 1),

where:

*Iin*—pixel intensity before TMO,

*Iout*—pixel intensity after TMO.

The application of the TMO formula for enhancing diagnostic details in the apical region in echocardiography videos required modification to achieve optimal results. We further refined this approach by introducing a parameter “*A*” in place of the constant “1” in the original TMO formula. This modification provides greater flexibility and control over the tone mapping transformation process.

The modified formula is expressed as:

*Iout* = *Iin*/(*Iin* + *A*)

where:

*Iin*—represents the pixel intensity before the TMO transformation.

*Iout*—represents the pixel intensity after the TMO transformation.

*A* is the introduced parameter, influencing the contrast of the resulting image. When *A* < 1, the image exhibits higher contrast, whereas when *A* > 1, the image demonstrates lower contrast.

The default value for the parameter “*A*” is set to 1. However, for each image, we adjust “*A*” empirically to optimize the image quality. If “*A*” is set to a value less than 1, the image gains more contrast, which may enhance the clarity of finer details. Conversely, if “*A*” is set above 1, the image becomes less contrasted. This flexibility allows for tailoring the contrast to each individual image, depending on the characteristics of the echocardiography machine and patient-specific factors, such as signal noise or anatomical variability. We found this empirical adjustment necessary to account for the inherent differences in image quality across echocardiograms.

Before applying this modified formula, pixel intensity values are first converted from the integer range of [0, 255] to the floating-point range of [0, 1]. This conversion ensures consistency in pixel intensity representation throughout the tone mapping process. The resulting pixel intensities remain within the floating-point range of [0, 1]. The resulting videos can be directly displayed on high dynamic range monitors. Alternatively, they can be converted back to the integer range of [0, 255] for display on standard monitors, as utilized in our investigation.

### 2.5. Simplified Explanation of the Left Ventricular Thrombus Detection Method (LVTDM)

The Left Ventricular Thrombus Detection Method (LVTDM) is a technique designed to improve the visibility of potential thrombi in the left ventricle using echocardiography videos. This method allows to obtain clearer images without the need for contrast agents, which are often used in traditional imaging but can pose risks to some patients. LVTDM employs a tone mapping technique, which is similar to enhancing photos so that they look better on regular screens. This technique helps convert images with limited details into clearer ones, making it easier to spot blood clots. The method focuses on the apical region of the left ventricle where blood clots are likely to form. By specifically improving the visibility of this area, LVTDM enhances the chances of detecting clots that might otherwise be missed. The process selectively increases the visibility of mid-tone shades in the apical area while maintaining overall image quality. This way, important details can be seen without losing clarity in other parts of the image. The method includes a formula to adjust image contrast, allowing for better adaptation to each patient’s specific needs. Once the images are processed, they can be displayed on various monitors, ensuring accurate assessment for thrombi and interpretation of results.

### 2.6. Statistical Methods

The IBM SPSS Statistics for Windows, Version 28.0 (Armonk, NY, USA: IBM Corp.) was used for statistical analysis.

For intergroup comparison of results Chi-Square test and Friedman test were used. The Friedman test was chosen for its appropriateness in handling our study design involving multiple related groups (the results of two observers’ assessments against the results from contrast echocardiography), its robustness to non-normal distributions, its ability to effectively evaluate intra-observer variability. Positive and negative predictive value, sensitivity and specificity were analyzed. *p*-value less than 0.05 were accepted as significant.

### 2.7. Ethical Approval

This retrospective study was conducted using patients’ files and formal approval by the Ethics (Helsinki) Committee at Shamir (Assaf Harofeh) Medical Center for the conduct of clinical studies was not required, nor was informed consent. Additionally, all images presented in the article are anonymous to ensure patient confidentiality. All methods were carried out following relevant guidelines and regulations.

## 3. Results

**Figure 1:**
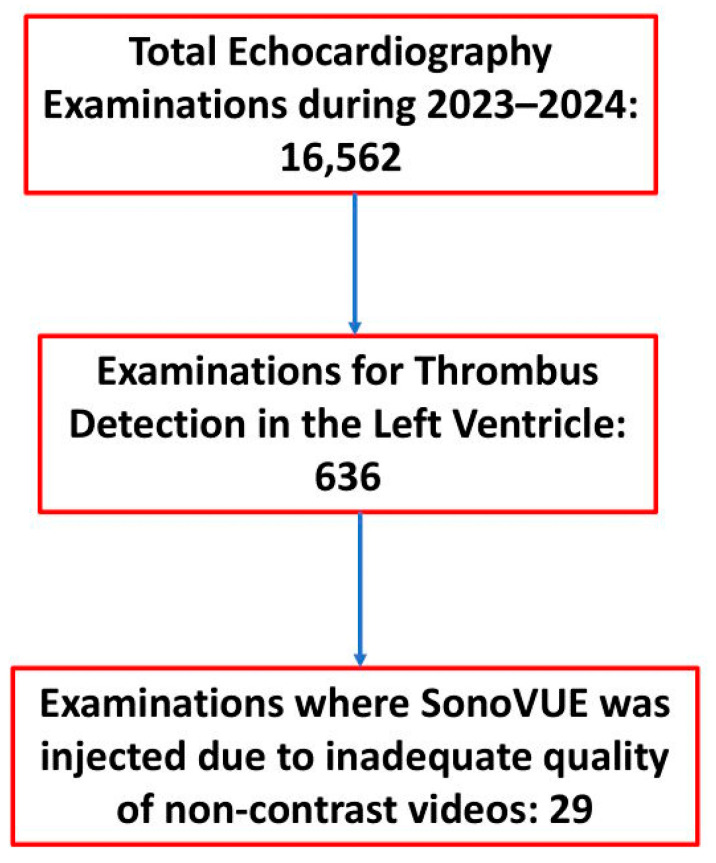
Patient Selection Flowchart. A total of 16,562 echocardiography examinations were performed in our echocardiography unit from 2023 to 2024. Among these, 636 examinations were conducted specifically to assess for thrombus in the left ventricle. In 29 of these cases, the echocardiography contrast agent SonoVue was injected due to insufficient video quality.

During the study period, contrast injection was performed in 29 patients to diagnose thrombus in the left ventricle (Figure 1). The demographic and clinical characteristics of these patients, along with the indications for their echocardiography examinations, are summarized in Table 1. The mean age of the patients was 63.4 ± 12.9 years, with 24 males. Echocardiography was performed in 16 patients in the setting of recent/acute anterior myocardial infarction, while 10 patients had a history of old anterior myocardial infarction. Additionally, one patient presented with hypertrophic apical cardiomyopathy accompanied by apical aneurysm, another was diagnosed with Takotsubo cardiomyopathy and apical akinesis, and one patient exhibited dilated cardiomyopathy with severe left ventricular dysfunction and apical akinesis. The mean ejection fraction was 39.6 ± 9.8%. All patients displayed apical wall motion abnormalities, with thrombus detected in 11 cases (37.9%). The average size of the thrombus was 1.6 ± 0.8 × 1 ± 0.3 cm.

In all patients, the primary echocardiography diagnosis was made following analysis by both the sonographer and echocardiography physician. The decision to administer SonoVue was based on the suboptimal image quality observed during conventional echocardiography examinations.

**Figure 2:**
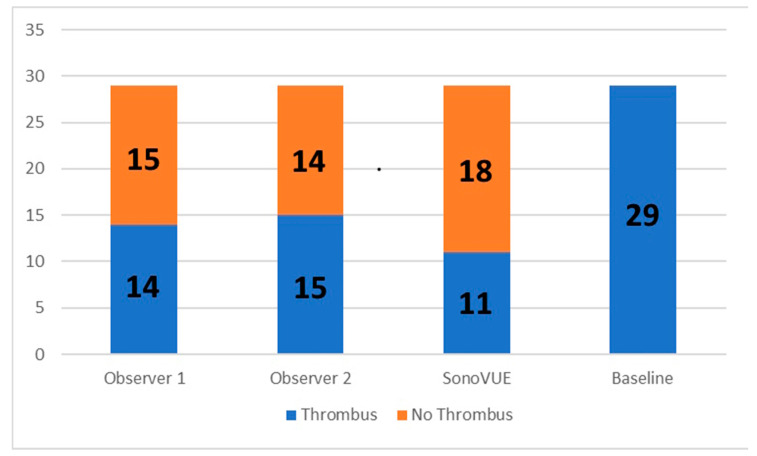
Diagram illustrating the results obtained by two independent observers using LVTDM, contrast-echo, and non-contrast transthoracic echocardiography. The figure depicts the number of cases in which thrombus was detected or not detected after postprocessing by observer 1, observer 2, injection of ultrasonic contrast SonoVue, and at baseline images. All baseline images were suspicious for thrombus. After postprocessing with LVTDM, thrombus was identified by observer 1 in 14 cases and by observer 2 in 15 cases. Thrombus was detected in 11 patients using SonoVue. Overdiagnosis of thrombus detection occurred in 3 cases for observer 1 and 4 cases for observer 2 after LVTDM analysis. Thrombus was not missed with LVTDM by either observer 1 or observer 2.

The results of the blind analysis conducted by two highly qualified observers (ML and VT) are presented in Figure 2 and Table 2, comparing the assessments with videos obtained after SonoVue injection.

After postprocessing with LVTDM, thrombus was observed by observer 1 in 14 cases (48.3%) and by observer 2 in 15 cases (51.7%). With the use of SonoVue, thrombus was detected in 11 patients. Observer 1 over-diagnosed thrombus detection with LVTDM in three cases, while observer 2 did so in four cases. No instances of missed thrombus diagnosis were observed with LVTDM, as shown in Figure 2.

**Figure 3:**
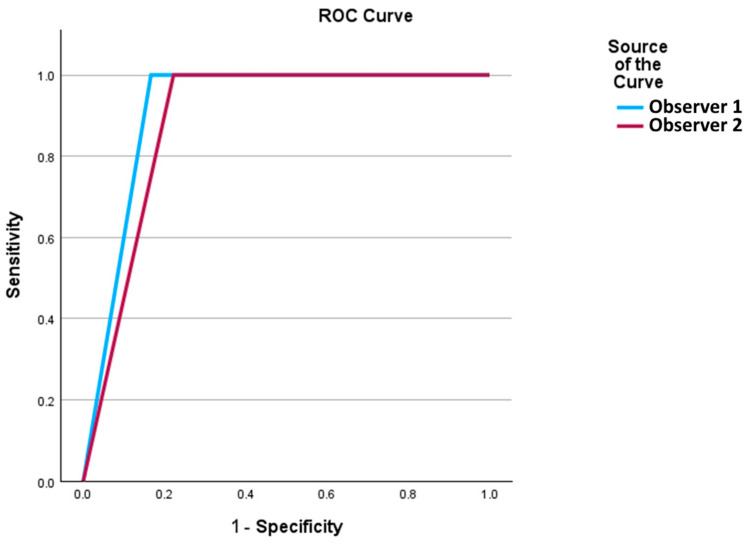
Diagnostic performance of the LVTDM compared to contrast echo results, assessed using Receiver Operating Characteristic (ROC) curves. The diagnostic performance of the Left Ventricular Thrombus Detection Method (LVTDM) compared to contrast echocardiography results, assessed using ROC curves. The area under the ROC curve (AUC) for Observer 1 was 0.917, while for Observer 2, it was 0.889. Observer 1 demonstrates a higher discriminatory power compared to Observer 2, as indicated by its higher AUC value of 0.917 versus Observer 2’s AUC of 0.889. Both observers achieved AUC values greater than 0.5, suggesting high diagnostic accuracy.

The sensitivity and specificity of LVTDM were 100% and 83%, respectively. The positive and negative predictive values of LVTDM were 79% and 100%, respectively, with an accuracy of 90%, as indicated in Table 3 and Figure 3.

**Figure 4:**
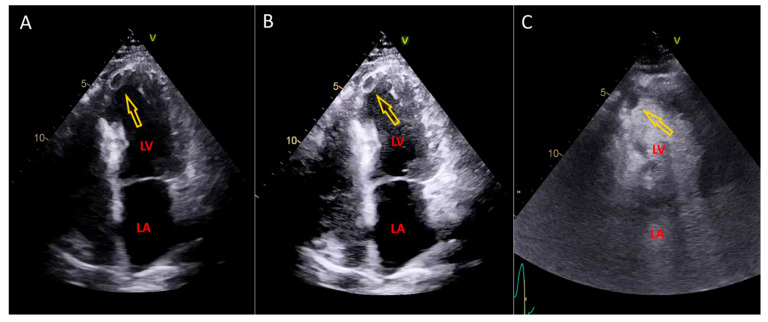
Echocardiography images depicting thrombus in the left ventricle obtained by conventional echocardiography, after postprocessing with LVTDM, and contrast echo. (**A**). Conventional echocardiography image showing suspicion of thrombus in the apex of the left ventricle [arrow], requiring confirmation by additional methods. (**B**). Image obtained after postprocessing with LVTDM, providing improved visualization of thrombus in the left ventricle [arrow]. (**C**). Contrast echocardiography image following injection of SonoVue. A filling defect in the apex of the left ventricle is consistent with thrombus [arrow]. Abbreviations: LV—left ventricle, LA—left atrium.

An example of an image that underwent postprocessing with LVTDM, in comparison with contrast echo, is presented in Figure 4, Videos S1–S3.

## 4. Discussion

Our study demonstrated a strong correlation between LVTDM applied on conventional echo videos and contrast echo, with a high sensitivity of 100% and specificity of 83%. The overall accuracy of the LVTDM was 90%. Notably, thrombus in the left ventricle was not missed by LVTDM; however, overestimation of thrombus may occur. Importantly, if no thrombus was found by LVTDM, contrast injection is not necessarily warranted due to the high negative predictive value of the method.

Various technologies were employed for thrombus detection, including multimodality imaging such as cardiac magnetic resonance (CMR), echocardiography with and without contrast, and a dual isotope subtraction method utilizing ¹¹¹In-platelet scintigraphy and 99mTc-blood pool scintigraphy for diagnosis [8,9]. Additionally, left ventriculography and multidetector-row computed tomography (CT) were utilized for thrombus diagnosis in the left ventricle [10]. Studies have shown that CT scans provide comparable specificity and sensitivity to two-dimensional transthoracic echocardiography (TTE) in identifying left ventricular thrombus [11].

Cardiac MRI is widely acknowledged as the premier non-invasive imaging modality for diagnosing thrombus [12,13,14], boasting a sensitivity ranging from 82% to 88% and a specificity of up to 100% when compared with surgical and pathological confirmation [15].

Among echocardiographic techniques, contrast echocardiography shows a strong correlation with cardiac magnetic resonance (CMR) in thrombus diagnosis, with a statistical significance of *p* < 0.001 [6]. Older studies report that the sensitivity of non-contrast transthoracic echocardiography for detecting left ventricular thrombus ranges from 21% to 35% [16,17]. However, a recent meta-analysis of studies from the past decade reported the sensitivity and specificity of non-contrast transthoracic echocardiography to be 47% and 98%, respectively, when compared with CMR [18]. In a more recent study [19], the sensitivity and specificity of TTE for detecting left ventricular thrombus were reported as 71% and 100%, respectively, using contrast echocardiography as the reference. Despite its limitations, echocardiography remains invaluable, as TTE is the only technique capable of providing dynamic, real-time bedside imaging of the heart [20]. Additionally, echocardiography speckle tracking imaging [21] can be useful in detecting underlying cardiac pathology and differentiating between regional left ventricular dysfunction, which is characteristic of ischemic heart disease, and the diffuse wall motion abnormalities more commonly seen in non-ischemic cardiomyopathy. While CT and MRI are indeed more precise, they are also more expensive, time-consuming, and less accessible. LVTDM provides a rapid, cost-effective solution for routine clinical practice, particularly for patients who are unable to undergo contrast-enhanced studies or MRI.

All of the previously mentioned studies did not classify echocardiography examinations based on image quality, which is crucial for accurate thrombus diagnosis. In our study, contrast was selectively used to enhance visualization of the endocardial border only in cases of suboptimal transthoracic echocardiograms. According to the most recent guidelines [1], echocardiography remains the first-line imaging test for detecting LV thrombus. In cases where the apex is poorly visualized on standard echocardiography, contrast echocardiography may be considered to improve image quality, while CMR should be considered for patients with equivocal echocardiographic images. Indeed, CMR is a gold standard for cardiac thrombus diagnosis [22].

Our findings suggest that the proposed LVTDM has a high negative predictive value and can significantly reduce the number of non-diagnostic echocardiography examinations, potentially by half.

**Figure 5:**
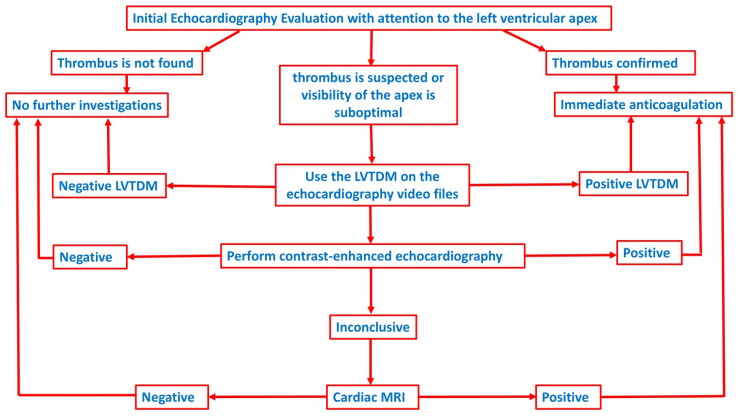
Proposed Diagnostic Algorithm for Left Ventricular Thrombus Detection. LVTDM is particularly valuable in routine echocardiographic exams where image quality or apex visualization is compromised, offering a sensitive method for detecting thrombi early. Contrast echocardiography should be employed if image quality is still inadequate or if LVTDM results are positive. Cardiac MRI should be reserved for confirmatory purposes, especially in cases where treatment decisions hinge on thrombus presence.

Overdiagnosis of thrombus may lead to unnecessary anticoagulation therapy or further diagnostic testing. We propose a thrombus detection algorithm, illustrated in Figure 5, in which LVTDM is utilized when conventional echocardiography results are inconclusive. To prevent overdiagnosis, contrast echocardiography should be employed at the next step. In non-diagnostic cases, MRI should be utilized as a confirmatory study.

### Limitations

The retrospective nature of our study could introduce certain biases, such as selection bias or incomplete datasets. Future prospective studies are needed to confirm the findings.

While the study was conducted by experts, future research could explore inter-observer variability among less experienced operators to assess the method’s generalizability.

In this study, our method was validated against contrast echocardiography. A larger study validating the method with CMR is desirable.

In this study, we focused on the detection of thrombus in the apical region. The applicability of our method to other possible thrombus localizations also requires verification and further investigation.

In conclusion, our study introduced LVTDM as a novel method to enhance diagnostic accuracy in echocardiography, particularly in cases of suboptimal image quality. By leveraging the rich detail present in echocardiography videos, LVTDM amplifies diagnostic efficacy, potentially reducing the need for contrast injection and providing a simpler, cost-effective approach to echocardiography image analysis. These findings suggest the potential for integrating LVTDM into routine clinical practice, thereby improving the accessibility and efficiency of echocardiography imaging. While the initial results are promising, larger studies are needed to validate the findings and establish the method’s diagnostic value more broadly in larger, confirmatory studies.

## Figures and Tables

**Table 1:** Clinical data of 29 patients that underwent contrast echocardiography for diagnosis of thrombus.

Total Number of Patients	*n*	29
Mean Age, years	mean ± SD	63.4 ± 12.9
Male	*n* (%)	24 (82.8%)
Female	*n* (%)	5 (17.2%)
*Indications for echocardiography*		
Acute/Recent myocardial infarction	*n* (%)	16 (55.2%)
Old myocardial infarction	*n* (%)	10 (34.5%)
Takotsubo Cardiomyopathy	*n* (%)	1 (3.4%)
Hypertrophic Apical Cardiomyopathy with aneurysm	*n* (%)	1 (3.4%)
Dilated Cardiomyopathy	*n* (%)	1 (3.4%)
Ejection Fraction, %	mean ± SD	39.6 ± 9.8
Apical wall motion abnormalities	*n* (%)	29 (100%)
Thrombus was found	*n* (%)	11 (37.9%)
Size of thrombus, cm	mean ± SD	1.6 ± 0.8 × 1.0 ± 0.3

**Table 2:** Friedman Test and Chi-Square test statistics for assessment of correlation between two observers and between contrast echo.

Ranks	Mean Rank	Comparisons	*p*-Value	N	29
				Chi-Square	4.333
Observer 1	2.03	Intra-observers	0.996	df	2
Observer 2	2.09	Observer 1 vs. contrast echo	0.9997	Asymp. Sig.	0.115
Contrast echo	1.88	Observer 2 vs. contrast echo	0.993	Friedman test	

df—degrees of freedom associated with the chi-square value, Asymp. Sig.—asymptotic significance value = *p* value, N—number of observations.

**Table 3:** Echocardiographic thrombus detection performance metrics table.

PPV	NPV	Accuracy	Sensitivity	Specificity
79%	100%	90%	100%	83%

PPV—positive predictive value, NPV—negative predictive value.

## Data Availability

The data are available on a reasonable request.

## Supplementary Materials

The following supporting information can be downloaded at: https://www.mdpi.com/article/10.3390/medicina60111815/s1, Video S1. Conventional echocardiography image showing suspicion of thrombus in the apex of the left ventricle, requiring confirmation by additional methods; Video S2. Image obtained after postprocessing with LVTDM, providing improved visualization of thrombus in the left ventricle; Video S3. Contrast echocardiography image following injection of SonoVue. A filling defect in the apex of the left ventricle is consistent with thrombus.
